# The relationship between rigorous perception of one’s own body and self, unhealthy eating behavior and a high risk of anorexic readiness: a predictor of eating disorders in the group of female ballet dancers and artistic gymnasts at the beginning of their career

**DOI:** 10.1186/s40337-022-00574-1

**Published:** 2022-04-11

**Authors:** Magdalena Leonkiewicz, Agata Wawrzyniak

**Affiliations:** Department of Human Nutrition, Institute of Human Nutrition Sciences, Warsaw University of Life Sciences (WULS-SGGW), Warsaw, Poland

**Keywords:** Perception of one’s own body and self, Eating behavior, Risk of anorexic readiness, Ballet dancers, Artistic gymnasts

## Abstract

**Background:**

A group that is particularly exposed to eating disorders are young sportswomen who practice aesthetic disciplines, for whom it is important to keep a slim figure. Hence, the purpose of the study was to present the assessment of perception of one’s own body and self as well as nutritional behavior in the group of ballet dancers and artistic gymnasts (BGA, n = 60) aged 10–12 against the background of the peer group (K, n = 60) and to determine the relationship between the studied elements, as well as anorexic readiness risk assessment to help diagnose premorbid stage of eating disorders with full symptoms.

**Methods:**

Anthropometric measurements (height, body weight) and the assessment of adipose tissue were performed. Using a questionnaire, data on physical activity, perception of one's own body and self, and selected eating behaviors were collected.

**Results:**

Underweight was observed in nearly half of the girls from the BGA group and the content of adipose tissue was significantly lower. Girls from the BGA group were characterized by overestimation of body size (*p* = 0.032), the need to improve their appearance/body (*p* = 0.025) and wanting to be the best in many areas of life (*p* = 0.002) significantly more often than in the K group. Moreover, they significantly more often limited the consumption of fats and carbohydrates (*p* = 0.044) and felt angry with themselves after too large of a meal (*p* = 0.050). It was shown that unhealthy eating behavior in the BGA group was significantly associated with rigorous self-perception (r = 0.42; *p* < 0.001). Students from the BGA group were more often exposed to a higher risk of anorexic readiness (*p* = 0.001). In a detailed analysis, it was found that eating behaviors, such as fasting, limiting the consumption of fats and carbohydrates, and avoiding eating under stress, were associated with feelings of dissatisfaction with oneself, the belief that appearance is extremely important in achieving life success and the need to improve appearance.

**Conclusion:**

The obtained research results can be used as a source of information for specialists (including dietitians and psychologists), for the preparation of educational and repair programs in the group of ballet dancers or artistic gymnasts aged 10–12, including nutritional education and psychological care.

**Supplementary Information:**

The online version contains supplementary material available at 10.1186/s40337-022-00574-1.

## Plain English summary

There is a risk that rigorous self-perception may promote unhealthy eating behavior and, consequently, have a negative effect on the nutritional status, as well as pose a risk of developing eating disorders. A group that is particularly exposed to eating disorders are young sportswomen who practice aesthetic disciplines, for whom it is important to keep a slim figure. Hence, the purpose of the study was to present the assessment of perception of one’s own body and self as well as nutritional behavior in the group of ballet dancers and artistic gymnasts aged 10–12 against the background of the peer group, as well as anorexic readiness risk assessment. In a detailed analysis, it was found that incorrect eating behaviors, such as fasting, limiting the consumption of fats and carbohydrates, and avoiding eating under stress, were associated with feelings of dissatisfaction with oneself, the belief that appearance is extremely important in achieving life success and the need to improve appearance.

The obtained research results can be used as a source of information for specialists (including dietitians and psychologists), for the preparation of educational and repair programs in the group of ballet dancers or artistic gymnasts aged 10–12, including nutritional education and psychological care.

**Supplementary Information:**

The online version contains supplementary material available at 10.1186/s40337-022-00574-1.

## Background

Correct eating behavior is one of the factors that promote health [1]. They are formed in childhood and may have an impact on health in adulthood. There are many factors that can influence the nutritional behavior of children and adolescents [2] including the self-esteem of young people, i.e. the perception of one’s own body and self. There is a risk that rigorous self-perception may promote unhealthy eating behavior and, consequently, have a negative effect on the nutritional status, as well as pose a risk of developing eating disorders, which usually begin in the early adolescence period and may reveal or intensify later [3–5]. The reason for the occurrence of eating disorders and as a consequence of psycho-physical exhaustion of young people, is, among others, excessive restriction of food consumption in order to reduce body weight. Such behaviors are to allow young people, in their opinion, to control their body and psyche [5]. A group that is particularly exposed to eating disorders are young sportswomen who practice aesthetic disciplines (including artistic gymnastics or figure skating), for whom it is important to keep a slim figures. This group also includes dancers, among others ballet, due to the similar specificity of these discipline [3, 6–10]. The need to maintain a certain body weight is often associated with good sports results or a favorable aesthetic impression; however, too low of a body weight and adipose tissue content negatively affect sports performance [11].

Low availability of energy from the diet, disorders of the menstrual cycle and reduction of bone mineral density is a premise for the diagnosis of the full form of the “Female Athlete Triad” which most often concerns women practicing sports competitively (currently defined as Relative Energy Deficiency in Sport (RED-S) to reflect that it also occurs in males and affects more than the 3 areas of health originally described) [12, 13]. The health effects of the female athlete triad may be long-term, including for the development of the skeletal or endocrine system [13–15]. Therefore, there is a need to undertake actions aimed at verifying whether rigorous perception of one’s own body and self in the group of artistic gymnasts and ballet dancers can promote, and if so, to what extent, inappropriate eating behavior during their initial professional career.

Studies on the assessment of nutritional behavior or nutritional status in the group of ballet dancers or artistic gymnasts are sparse [16–21] and so far have been conducted mainly among older ballet dancers, over 14–18 years of age or adults [8, 9], and not among younger girls, who are at the beginning of their artistic career. No studies have been undertaken to identify factors that may influence eating behavior in the group of ballet dancers or artistic gymnasts aged 10–12, which is one of the key elements of understanding the possible causes of eating disorders. Therefore, the authors of the study hypothesized that the group of girls professionally practicing artistic gymnastics and ballet in a professional school (participating in professional opera and ballet performances and in artistic gymnastics competitions) is characterized by a greater intensity of improper eating behavior than their peers, which is associated with a rigorous perception of their own body and self at the beginning of their professional career. Due to the very young age of the participants, the risk of anorexic readiness was studied, the higher level of which may be a predictor of eating disorders. Anorexic Readiness Syndrome (ARS) has been defined as a group of indicators located in the cognitive, emotional and behavioral spheres of functioning and confirming the occurrence of unhealthy eating behavior and a rigorous perception of one's own body and self. The higher level of ARS does not determine the occurrence of anorexia nervosa, but it may indicate inclination to the so-called anorectic behavior. The term ARS was created by the Polish researcher [22].

The obtained research results will be used as a source of information for specialists (including dietitians and psychologists), for the preparation of educational and repair programs in the group of ballet dancers or artistic gymnasts aged 10–12, including nutritional education and psychological care.

## Methods

### General information

The presented results are a component of the initial part of the project (experiment), the broader aim of which was to assess the impact of nutritional education on changes in the diet and nutritional status of students from the ballet school and artistic gymnastics classes in a 6-month period and the experiment was primarily of a nutritional nature. The diagram presenting the overall activities within the project is provided in the Additional file 1: Figure 2.

The article presents the results of perception of one’s own body and self as well as selected nutritional behaviors of ballet dancers and gymnasts against the background of the peer group collected in the first part of the experiment. The research was conducted by a person with a university degree in human nutrition, with many years of experience in conducting this type of research. On January 7th, 2015, the consent of the Bioethics Committee at the Food and Nutrition Institute in Warsaw, written consent of school heads for the participation of students in the study, written consent of the legal guardians of the respondents, and the consent of the respondents themselves, were obtained for the study. The research was conducted in accordance with the ethical principles contained in the Declaration of Helsinki. The students and their legal guardians were informed about the purpose of the study and were acquainted with its detailed program.

### Study participants

The study was conducted among 60 female students of the state ballet school and artistic gymnastics classes (professionally practicing ballet and artistic gymnastics), aged 10–12 (BGA group) and 60 primary school female students as a control group (K group), of the same age and from the same urban centers. Ballet dancers and gymnasts were classified in the same group due to the similar features and the similar nature of ballet and artistic gymnastics. The criteria for exclusion from the study were the presence of a chronic disease in the students, e.g. diabetes, physical inactivity related to the current injury or disease, lack of consent of the legal guardian or the child to participate in the study.

### Assessment of nutritional status and physical activity

To assess the nutritional status of girls from the BGA and K groups, anthropometric measurements (body weight, height) were used, as well as the content of adipose tissue in the body was assesed. Body weight was measured in accordance with the procedure using a TANITA BC 730 WH 36 electronic scale (Tanita Corporation, Tokyo, Japan), growth was measured with a SECA 213 height measuring device (Seca, Hamburg, Germany) [23]. Based on the measurements of height and weight, the body mass index (BMI) in kg/m^2^ was determined, which was compared with the reference values of percentile grids for girls aged 7–18, developed in the nationwide OLAF project for the Polish population [24]. Percentile values for BMI read from the percentile grids were assessed with the following criteria: underweight < 5th percentile, normal body weight 5–85 percentile, overweight > 85 and ≤ 95 percentile, and obesity > 95 percentile. On this basis, the number and percentage of girls with underweight, normal body weight and overweight were determined in both groups. The test of adipose tissue content was performed using the bioimpedance method (BioScan 920-2 device, Maltron International), with the use of 4 disposable electrodes (according to the procedure). The content of adipose tissue (measured as a percentage) was related to the reference value established for girls, taking the minimum value at the level of the 3rd percentile for the age of 10–12 years, which was 14–15% [25].

The study assessed the duration of physical activity, divided into school and out-of-school physical activity. Girls’ physical activity during school time was determined on the basis of the program at school for children aged 10–12 in a given type of school, which was 10–12 lessons per week for ballet dancers and gymnasts and 4 lessons per week for the control group. One lesson lasted 45 min. The number of hours of out-of-school physical activity was determined on the basis of a question from a questionnaire addressed to girls, which concerned the types of additional sports activities undertaken by the respondents and their duration during the day. Activities were assessed separately in the test groups and also after summation as full clock hours per week.

### Perception of one’s own body and self

Assessment of the perception of one's own body and attitude towards oneself was made with the use of 8 dichotomous questions (requiring “yes” or “no” answers), using an adaptation of the method for assessing the level of anorexic readiness syndrome (ARS) by Ziółkowska [22], which is commonly used in Poland. This method determines the predisposition to the occurrence of eating disorders and has been used in studies among various groups of adolescents and adults [26–30]. A pilot study was conducted among 12 girls from the BGA group and 12 girls from K group to verify the questions. All comments were taken into account when creating the final version of the questionnaire. Our questionnaire contained 6 questions from the ARS method. They asked whether the respondents: have a sense of proportionality of their own body, control body weight and dimensions, have a feeling that appearance is of great importance in achieving success in life, would like to improve their appearance/body, often feel depressed and dissatisfied with themselves, they have a need to be the best in many areas. The above assessment was supplemented with 2 original questions. The first was whether the girls believed that their body weight was normal, too low or too high in relation to age-appropriate body weight of their peers (not practicing artistic disciplines). The responses were related to the actual body weight of the respondents, and on this basis, it was assessed whether there is a overestimation of body size. The second original question was whether girls feel dissatisfied with their body weight. For each answer indicating disturbed self-esteem, assessed using the ARS questionnaire and original questions, 1 point was awarded. The greater the number of points obtained (max. 8), the more rigorous was the perception of one's own body and self. The results were summarized by presenting the average number of points obtained for each group, divided into categories taking into account the severity of rigorous perception of one's own body and self. In the case of obtaining ≤ 3 points, the rigor of self-assessment was assessed as lower, for 4–6 points as medium, and for 7–8 points as higher.

### Assessment of eating behavior

Selected eating behaviors were assessed using 8 dichotomous questions (requiring “yes” or “no” answers), 5 of which were from the anorexic readiness syndrome measure ARS [22]. The questionnaire asked whether the respondents: were on fasting or slimming diets, limited the consumption of fats and carbohydrates, became angry with themselves after the large meal, took weight loss or appetite-suppressants, and took laxatives. The above assessment of nutritional behavior was supplemented with 3 original questions concerning: avoiding eating in stressful situations, eating less than 5 meals a day, and eating less than 3 meals a day (a more rigorous version of the previous question). For each incorrect eating behavior, assessed using the ARS questionnaire and original questions, 1 point was awarded, and the greater the number of points obtained (max. 8), the greater the irregularity of the assessed eating behavior. The results were summarized by presenting the average number of points obtained for each group, divided into categories taking into account the severity of unhealthy eating behavior. In the case of obtaining ≤ 4 points, the severity of unhealthy nutritional behavior was assessed as lower, for 5–6 points as medium, and for 7–8 points as higher.

### Anorexic readiness risk assessment

To assess the level of anorexic readiness, the points obtained for responses in the study perception of one's own body and self and assessment of eating behavior were summed up, as recommended by the author of the ARS method [22]. The greater number of points obtained by the respondents (max. 16), indicated a greater risk of anorexic readiness. In the case of obtaining ≤ 6 points, the risk was assessed as low, for 7–11 points as medium, and for 12–16 points as high.

### Statistical analysis

Statistical analysis was performed using the statistical program Statistica 13.0 (StatSoft Polska). For quantitative variables, the mean and standard deviations were calculated and ranges (min–max) were determined. In order to check the compliance with the normal distribution, the Shapiro–Wilk test was used. Quantitative data such as age, weight, height, BMI, body fat content and number of hours of physical activity per week were analyzed with the Mann–Whitney U test for data with an abnormal distribution. Categorical variables, such as eating behavior or perception of one's body and self were compared with the Pearson Chi^2^ test, and Wald's test was also used to determine the risk of a given abnormality in the BGA group compared to the K group, for which the odds ratio was set at OR = 1. The summary of the points obtained in the assessment of body and self-perception and in the assessment of eating behavior was compared between the groups with the Mann–Whitney U test (for means) and the Pearson Chi-square test (for categories). The size of Cohen's d effect was also calculated for the groups (d < 0.5 small effect, 0.5 < d < 0.8 medium effect, d > 0.8 large effect). In the BGA group, the Spearman correlation test was used to determine the relationship between the perception of one's own body and self as well as the assessment of eating behavior. The BGA group also analyzed the correspondence between the components of body and self-perception and selected eating behaviors. The analysis took into account the nutritional behaviors and elements of body and self-perception for which OR > 1 was observed for the BGA group compared to the K group (Tables 2, 3). Only the following components were omitted for the condition OR > 1: intake of weight loss or appetite suppressants and taking laxatives, due to the fact that they concerned a small percentage of respondents, and consumption of < 5 meals a day, because the more stringent version of limiting the amount was selected for the analysis meals during the day < 3. The analysis included the factor of frequent depressed mood and self-dissatisfaction (although OR < 1 was observed in the BGA group) due to its importance and indicating it as a factor influencing improper eating behavior, leading to the development of eating disorders, which may be used by other authors in future research [31, 32]. The level of statistical significance for the evaluation of the results was set at α = 0.05, and for *p* ≤ 0.1, the statistical trend was determined.

## Results

### Characteristic of group

The BGA group had a significantly lower body weight, on average by 6.5 kg (16%) compared to their peers attending traditional school (Table 1). Also, the mean body mass index (BMI) was significantly lower in the group of young female artists (on average by 2.1 kg/m^2^; i.e. by 12%), with the lowest value being 12.6 kg/m^2^. The nutritional status determined on the basis of percentile grids indicated the presence of underweight in nearly half of ballet dancers and artistic gymnasts. On the other hand, the nutritional status of their peers from K group showed a 10% were underweight and a 13% were overweight girls, which was not found among sportswomen. The content of body fat was significantly lower in the BGA group, and in 33% of ballet dancers and gymnasts, it was below the reference value (14–15% of adipose tissue) adopted for girls of this age [25].

**Table 1:** Characteristic of the BGA group and the K group

Factor	BGA n = 60		K n = 60		*p*
	Mean ± SD	Min–Max	Mean ± SD	Min–Max	
Age (years)	11.1 ± 0.7	10–12	10.9 ± 0.7	10–12	0.192*
Weight (kg)	34.8 ± 6.5	23.7–52.0	41.3 ± 9.7	26.1–63.2	< 0.001*
Height (cm)	147.6 ± 8.0	129.0–164.0	150.6 ± 10.1	136.0–172.5	0.173*
BMI (kg/m^2^)	15.9 ± 2.0	12.6–21.8	18.0 ± 2.7	13.8–25.9	< 0.0001*
Nutritional status (%)					
Underweight	43		10		< 0.001**
Normal body weight	57		77		
Overweight	0		13		
Average body fat content (%)	16.2 ± 3.5	7.9–24.9	18.2 ± 3.1	10.9–29.3	0.005*
Body fat content by percentile grids (%)					
Insufficient	33		10		0.003**
Acceptable	63		76		
Excessive	4		14		
Physical activity (number of hours/week)					
At school	8.7 ± 0.0	8.3–9.0	3.0 ± 0.0	3.0–3.0	< 0.001*
Out of school	4.2 ± 3.2	1.0–15.7	2.0 ± 2.2	0.0–7.0	< 0.001*
Total	12.9 ± 3.2	10.0–24.0	5.0 ± 2.2	3.0–10.0	< 0.001*

*The Mann–Whitney U test

**The Pearson’s chi-square test; *p* ≤ 0.05—statistical significance

Traditional school students devoted significantly less time (by approx. 60% compared to the BGA group) to physical activity during the week, both at school (almost 3 times less), due to the smaller number of physical activities provided for by the school program, as well as outside school (2 times less).

### Perception of one’s own body and self

In the BGA group, almost 1/4 expressed dissatisfaction with their body weight, even though overweight was not reported in this group (Table 2). Ballet dancers and artistic gymnasts were characterized by overestimation of body size significantly more often compared to the K group (*p* = 0.032), and to a much greater extent compared to their peers who did not train artistic gymnastics or ballet, expressed the need to improve their appearance/body (*p* = 0.025) and wanting to be the best in many areas of life (*p* = 0.002). Compared to the control group, young sportswomen were characterized by a higher odds ratio (OR = 2.769, OR = 3.267; *p* ≤ 0.05) for the occurrence of the latter beliefs. The total mean number of points obtained in the assessment of own body and attitude to oneself in the BGA group was significantly higher by 0.6 points compared to the average score of their peers who did not train (*p* = 0.047; Cohen’s d effect size = 0.491). This result may indicate a more rigorous self-esteem of young female athletes (Table 4).

**Table 2:** Perception of one’s own body and self in the BGA group and in the K group

Perception of one’s own body and self	BGA n = 60 (%)	K n = 60 (%)	*P* (Chi^2^)	OR for BGA (95% CI)
Overestimation of body size	25	18	0.032*	1.484 (0.612–3.602)
Dissatisfaction with body weight	22	18	0.581	1.232 (0.498–3.049)
Lack of a sense of proportionality of one's own body	27	35	0.323	0.675 (0.307–1.486)
Control body weight and dimensions	65	67	0.847	0.861 (0.399–1.854)
The belief that the appearance is of great importance in achieving life success	43	30	0.129	1.667 (0.778–3.569)
Need to improve appearance/body	37	18	0.025*	2.769* (1.190–6.442)
Feel depressed and dissatisfied with themselves	33	37	0.702	0.863 (0.404–1.844)
Wanting to be the best in many areas of life	70	42	0.002*	3.267* (1.525–6.997)

*p ≤ 0.05—statistical significance; the Pearson’s chi-square test or the Wald test (OR values)

### Assessment of eating behavior

Incorrect eating behavior was observed in both the BGA and K groups (Table 3). Ballet dancers and artistic gymnasts reduced the consumption of fats and carbohydrates (*p* = 0.044) and felt angry with themselves after too large meal (*p* = 0.050) significantly more often than their peers from traditional school. Girls from the BGA group, compared to the K group, were characterized by a higher odds ratio of the occurrence of these unfavorable eating behaviors (OR = 2.111 and OR = 2.317; *p* ≤ 0.05). A large proportion of girls (48.5% of female dancers and 40% of their peers) reacted to stressful situations by avoiding food. There were also cases of using laxatives in both the BGA and K groups and the use of appetite suppressants, but no statistically significant differences were found in this case for the groups. The total mean number of points obtained in the assessment of nutritional behavior in the BGA group was significantly higher by 0.55 points compared to the average score of students from primary school (*p* = 0.033; Cohen's d effect size = 0.439). The obtained results confirm the tendency towards higher irregularities in eating behavior in the group of ballet dancers and gymnasts (Table 4).

**Table 3:** Eating behavior in the BGA group and in the K group

Eating behavior	BGA n = 60 (%)	K n = 60 (%)	*p* (Chi^2^)	OR for BGA (95% CI)
The use of fasting or slimming diets	27	18	0.274	1.619 (0.673–3.896)
Limiting the consumption of fats and carbohydrates	55	37	0.044**	2.111** (1.008–4.419)
Getting angry with yourself after too large of a meal	32	17	0.050**	2.317* (0.962–5.580)
Taking weight loss or appetite suppressants	3	2	0.154	2.385 (0.548–5.972)
Taking laxatives	7	5	0.697	1.357 (0.285–6.441)
Avoiding eating in stressful situations	49	40	0.648	1.397 (0.676–2.890)
Eating < 5 meals a day	55	48	0.465	1.307 (0.632–2.698)
Eating < 3 meals a day	15	7	0.142	2.800* (0.816–9.606)

*p ≤ 0.1; **p ≤ 0.05—statistical significance; the Pearson’s chi-square test or the Wald test (OR values)

**Table 4:** Assessment of perception of one’s own body and self and nutritional behavior, as well as the risk of anorexic readiness in the BGA group and in the K group

Assessment	BGA n = 60		K n = 60		*p*	
	Mean ± SD	Min–max	Mean ± SD	Min–max		
*Perception of one’s own body and self*						
Amount of points (max. 8)	5.02 ± 1.26	2.0–8.0	4.43 ± 1.14	2.0–7.0	0.047*	
Cohen's d effect size = 0.491						
Rigorousness in the perception of one's own body and self [%]						
Lower	10%		22%		0.073**	0.080**
Medium	57%		60%			0.711**
Higher	33%		18%			0.061**
*Eating behavior*						
Amount of points (max. 8)	6.15 ± 1.11	3.0–8.0	5.60 ± 1.38	2.0–8.0	0.033*	
Cohen's d effect size = 0.439						
Severity of unhealthy eating behavior (%)						
Lower	10%		20%		0.093**	0.125**
Medium	48%		55%			0.583**
Higher	42%		25%			0.079**
*Anorexic readiness risk assessment*						
Amount of points (max.16)	11.17 ± 2.06	5.0–16.0	10.03 ± 1.98	4.0–15.0	0.001*	
Cohen's d effect size = 0.564						
Risk of anorexic readiness (%)						
Lower	3%		3%		0.013**	1.000**
Medium	52%		77%			0.004**
Higher	45%		20%			0.003**

*The Mann–Whitney U test, **The Pearson’s chi-square test; *p* ≤ 0.1—statistical tendency, *p* ≤ 0.05—statistical significance

### Anorexic readiness risk assessment

The total mean number of points obtained in the assessment of the risk of anorexia readiness in the BGA group was significantly higher by 1.14 points compared to the average score of students from primary school in the group K (*p* = 0.001; Cohen’s d effect size = 0.564) (Table 4). Almost half of the girls in the BGA group (45%) had a higher risk of anorexic readiness, which significantly exceeded the frequency of this parameter in K group (*p* = 0.003).

### Relationship between self-perception and eating behavior

The study showed a statistically significant correlation between the perception of one's own body and self and the assessment of eating behavior (r = 0.42; *p* < 0.001) in female athletes.

**Fig. 1:**
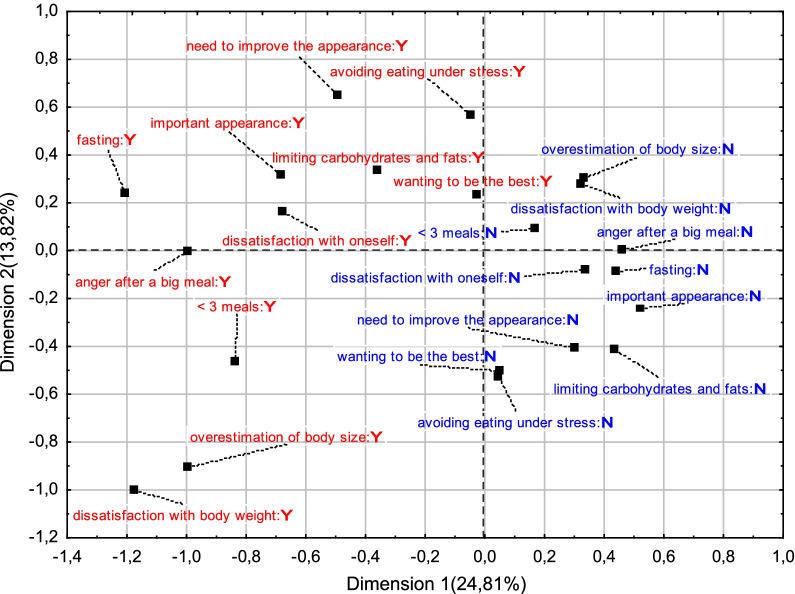
Correspondence analysis results of selected eating behaviors and body and self-perception components in the BGA group. Beneficial perceptions/behaviors are marked in blue, unfavorable in red; *Y* yes, *N* no

Based on the results of the correspondence analysis, it can be concluded that improper eating behaviors, such as fasting, limiting the consumption of fats and carbohydrates and avoiding eating under stress, were associated with the feeling of dissatisfaction with oneself, the belief that appearance is of great importance in achieving life success and the need to improve appearance (Fig. 1).

Eating less than 3 meals a day corresponded to dissatisfaction with body weight and a overestimation of body size. Beneficial eating behaviors such as eating more than 3 meals a day, not getting angry after a large meal were associated with normal body image and body weight satisfaction. In addition, the position that appearance does not matter much in terms of success, no need to improve appearance, no desire to be the best in many areas, and self-satisfaction corresponded to no restriction in carbohydrate and fat consumption, no fasting, and no avoiding eating in stressful situations.

## Discussion

The authors of the study confirmed the hypothesis that the group of girls aged 10–12 training in artistic gymnastics and ballet is characterized by a higher severity of unhealthy eating behavior than their peers from primary school, which is associated with a rigorous perception of their own body and self at the beginning of their professional career. In addition, students from the BGA group were more often exposed to a higher risk of anorexic readiness, which may predispose to the so-called anorexic behaviors.

### Body weight and adipose tissue content in the group of ballet dancers and artistic gymnasts

The period of childhood is the time of preparation of the organism to biological and psychological changes. Maintaining the correct body weight during this period is important for the proper growth and development of the body. The high percentage of underweight in the group of girls practicing aesthetic disciplines, observed in our study, complements the results of previous studies conducted in older age groups [10, 17, 18, 33, 34]. It also shows that the problem of underweight among ballet dancers and gymnasts already occurs at the age of 10–12 and increases with age and professional advancement. A similar relationship was also found in the study of the level of adipose tissue [20, 35].

Moreover, a study by Kostrzewa et al. [20] showed a greater difference in the content of body fat between the group of ballet dancers aged 13–14 years and the group of girls not training in aesthetic disciplines (14.5% vs. 28%). The content of body fat naturally increases with age, which is related to adolescence and fullness by adipose tissue, among others endocrine organ function [36, 37]. Too low (or even decreasing) level of adipose tissue may cause hormonal disorders such as menstrual disorders or delay in the onset of the first menstruation [17, 35, 38]. Therefore, it is important that the psychological and nutritional assessment of nutritional behavior in the group of ballet dancers and gymnasts begins as early as possible, even before adolescent, because, according to researchers, the acceptance of one's own body in the group of adolescent girls was clearly associated with a lower level of eating disorders [39]. Deficiency in body weight, especially significant and long-lasting, may be a diagnostic element of eating disorders, because the lack of weight gain in children leading to a BMI below the 5th percentile for age is one of the criteria for the diagnosis of anorexia according to the ICD-11 classification [40, 41].

### Threats resulting from the specific perception of one's own body and self in relation to nutritional behavior in the group of ballet dancers and artistic gymnasts

The profession of a ballet dancer and artistic gymnast requires maintaining a slim figure and low body weight, which is associated with the desire to achieve professional success [3, 21]. Durme et al. [10] reported a higher drive for thinness and more concerns about their weight and body shape in the group of girls training aesthetic disciplines compared to the general population. The adoration of low body weight, combined with the belief that body size can be changed, may result in a distortion of the perception of one's own body [42], and may also increase the risk of incorrect eating behavior and, consequently, eating disorders [8–10, 40]. Chen et al. [43] observed that the risk of trying to reduce body weight in the group of children and adolescents who overestimated it was 10 times higher compared to those who correctly assessed their body weight.

In our study, a lower percentage of girls with overestimation of body size and dissatisfaction with body weight was observed than in the studies of other authors in older age groups [3, 10, 18, 33]. The results of the self-assessment of body weight carried out among ballet dancers aged 14–18 showed that 55% of them considered themselves too fat, despite the fact that the average body mass index in this group was 18.56 kg/m^2^ [18]. Even greater disturbances in the perception of own figure were found in the group of artistic gymnasts aged 14 [33]. Although 88% of them were underweight, and as much as 37% were significant, only 3% of respondents noticed this irregularity. A high percentage of girls showing disturbances in the perception of their own figure was also found in the group of female students—models and ballet dancers, who showed a discrepancy between the calculated body mass index indicating the actual nutritional status and the self-assessment of the figure made by the respondents [21]. A study among women with anorexia nervosa showed that disturbed body image in this group is associated with top–down cognitive–affective distortion in evaluating their own body [44].

In the profession of a ballet dancer or gymnast, apart from the need to maintain a slim figure, persistence and accuracy, or even perfection and discipline in performing specific tasks are also important [3, 42]. In our study, almost 3/4 of the BGA group felt the need to be the best also in other areas, not only in the sphere of their profession. Girls who are perfectionists, like to compete and feel the need for success, most often develop neurotic perfectionism, which is characterized by excessively high expectations for oneself and others, and in the case of failure, low self-esteem appears [21, 45]. Thomas et al. [46] found that dancers with a high level of perfectionism may be at a higher risk of developing an eating disorder compared to dancers with a lower level of perfectionism or in a less competitive environment. And while perfectionism is not an abnormality in itself, higher level of perfectionism in the BGA group may be associated with the risk of developing an eating disorder, especially in young students with low body fat (below recommended values) and low body mass (BMI) and is also one of the factors included in Anorexic Readiness Syndrome assessment. On the other hand, our research showed that the lack of the need to be the best in many areas corresponded to the lack of avoiding eating during stressful situations. This may indicate that people with a lower level of perfectionism felt less psychological pressure and therefore did not limit their eating in stressful situations [9].

Another feature that can lead to inappropriate eating behavior, such as weight loss, is the lack of self-attractiveness [3, 47]. This is confirmed by the results of our study, which show the relationship between the belief that appearance is of great importance in achieving success and the need to improve appearance, with the use of fasting and limiting the consumption of fats and carbohydrates. The latter eating behavior was found in half of the respondents in the BGA group. These girls consciously avoided products rich in the above-mentioned macronutrients. Carbohydrates should be the main source of energy for the body, especially in the case of people with very high physical activity [48–50]. They should be found in the diet of a physically active person in an amount that complete the muscle glycogen stores, from which the body draws energy for physical exercise [49, 51–53], and which stores in young athletes run out faster than in adult players [54]. Too low carbohydrate intake by young dancers may not only lead to an energy deficit, but also reduce the body's efficiency during physical activity and lead to faster physical exhaustion [16, 49, 50]. Other examples of improper nutritional behaviors in order to reduce body weight and improve appearance were giving up certain types of products and reducing the number of meals during the day [18, 33]. The study by Gacek et al. [18] showed that 23% of 14–18-year-old ballet dancers consumed only 1–2 meals a day, and the results of our study show that eating < 3 meals a day in the BGA group corresponded to overestimation of body size of body weight, as well as dissatisfaction with body weight.

The fact of feeling angry with oneself after eating a large meal is also disturbing, which concerned twice the percentage of respondents in the BGA group compared to the K group. It was also observed by Chalcarz et al. [26], who found such behavior in 50% of girls from the group with average ARS level and in 100% of girls from the group with high ARS level, whose components are incorrect self-esteem and eating behavior. In our study, anger with oneself after eating a large meal corresponded with often depressed mood and dissatisfaction with oneself. Other authors indicate that self-criticism may be related to the need to be perfect in the eyes of others [32], and dissatisfaction with self is associated with eating behavior disorders [31].

Assessing ARS in the group of dancers aged 11–25, Chalcarz et al. [26] showed that the ARS score was medium in 62% of respondents, and high in nearly ¼. This means that most of the respondents showed moderate or high irregularity in self-esteem of their own body and person, as well as in eating behavior. The adaptation of the above method in our study allowed for obtaining equally disturbing results, indicating a greater tendency to rigorous perception of one's own body and self, and to incorrect nutritional behaviors in the BGA group, compared to the K group. This tendency was the most noticeable among girls with a large intensification of assessed irregularities. Research by other authors has shown that girls who feel insecure about their appearance are characterized by low self-esteem and the occurrence of eating disorders [39, 42].

The ARS assessment performed by other authors summarizes abnormalities in self-esteem and nutritional behaviors, but does not show the relationship between these elements. There are no studies in the literature in the group of ballet dancers and gymnasts in which the relationship between the perception of one's own body and self, and eating behavior would be assessed. The current study is the first innovative experiment to tackle this topic and to observe at the same time that unhealthy eating behavior is significantly related to rigorous self-perception. Therefore, it is necessary to intervene at the earliest possible stage of the professional development of this group in order to limit the occurrence of the consequences of the above-mentioned behaviors that are dangerous to health.

### Strengths and limitations

To our knowledge, no studies have been conducted to assess the causes of the eating behavior of ballet dancers and artistic gymnasts, including the relationship between the eating behavior in this group and the perception of their own body and self or to assess the risk of anorexic readiness. Another advantage of the study is that it was conducted among ballet dancers and gymnasts aged 10–12, who, as far as we know, have not been subjected to such a comprehensive study so far. The results of this study may therefore complement the studies of other authors conducted in other age groups and may prove helpful in creating nutritional education and psychological care in this group. The nutritional status was assessed on the basis of anthropometric measurements, and not on the basis of data on height and weight declared by the respondents. Measurements were carried out by a qualified dietitian, based on a standardized procedure.

A limitation of the study may be the small size of the BGA group. The selection of people and their number was influenced, among others, by the specificity of the profession performed by the respondents. Girls practicing ballet and artistic gymnastics constitute a narrow and sensitive group, among which, due to the presence of pro-anorexic behaviors, the selection of a larger number of people is extremely difficult to achieve, which is confirmed by the studies of other authors, which were also carried out in a few groups or slightly more numerous but heterogeneous groups by age of the respondents [6, 10, 17–21, 35, 56]. The size of the group also resulted from the fact that the study described in this work was part of a larger 6-month experiment, which was then repeated. Another limitation is a modification of the ARS questionnaire, which was adapted to the needs of the study of girls aged 10–12, taking into account factors important in the analysis of the relationship between the eating behavior and the perception of their own body and person. This modification was used to emphasize the importance of factors in the development of eating disorders as overestimation of body size, dissatisfaction with body weight as well as avoiding eating in stressful situations and the number of meals per day, which is an important element of the latest nutritional recommendations for children in Poland. Modifications as significant were added after analyzing the current scientific literature related to the topic.

## Conclusions

The results of the study confirmed that the BGA group was characterized by a greater incidence of unhealthy eating behavior, which was associated with a rigorous perception of one's own body and self at the beginning of the professional career, already at the age of 10–12, as well as a higher risk of anorexic readiness. This, in turn, may classify this group as a risk group for developing an eating disorder. Our results indicate the need for early nutritional or dietary intervention in the group of 10–12 year old young ballet dancers and artistic gymnasts, including nutritional education, e.g. in terms of consuming an appropriate number of meals during the day, the role of individual nutrients, the harmfulness of fasting and taking slimming. The intervention should also include psychological care in order to raise self-esteem, which affects eating behavior, health and job satisfaction.

## Supplementary Information

**Additional file 1**. Figure 2 Scheme of the project for assessing the impact of nutritional education on changes in the diet and nutritional status of ballet school students and artistic gymnastics classes.

## Data Availability

The datasets used and/or analysed during the current study are available from the corresponding author on reasonable request.
