# Supplementary material for: The relationship between rigorous perception of one’s own body and self, unhealthy eating behavior and a high risk of anorexic readiness: a predictor of eating disorders in the group of female ballet dancers and artistic gymnasts at the beginning of their career
Source: J Eat Disord. 2022 Apr 11;10:48. doi: 10.1186/s40337-022-00574-1 (PMC8996514; doi:10.1186/s40337-022-00574-1)
Supplement: Supplementary file 1 — Additional file 1. Figure 2 Scheme of the project for assessing the impact of nutritional education on changes in the diet and nutritional status of ballet school students and artistic gymnastics classes. [file 40337_2022_574_MOESM1_ESM.pdf]

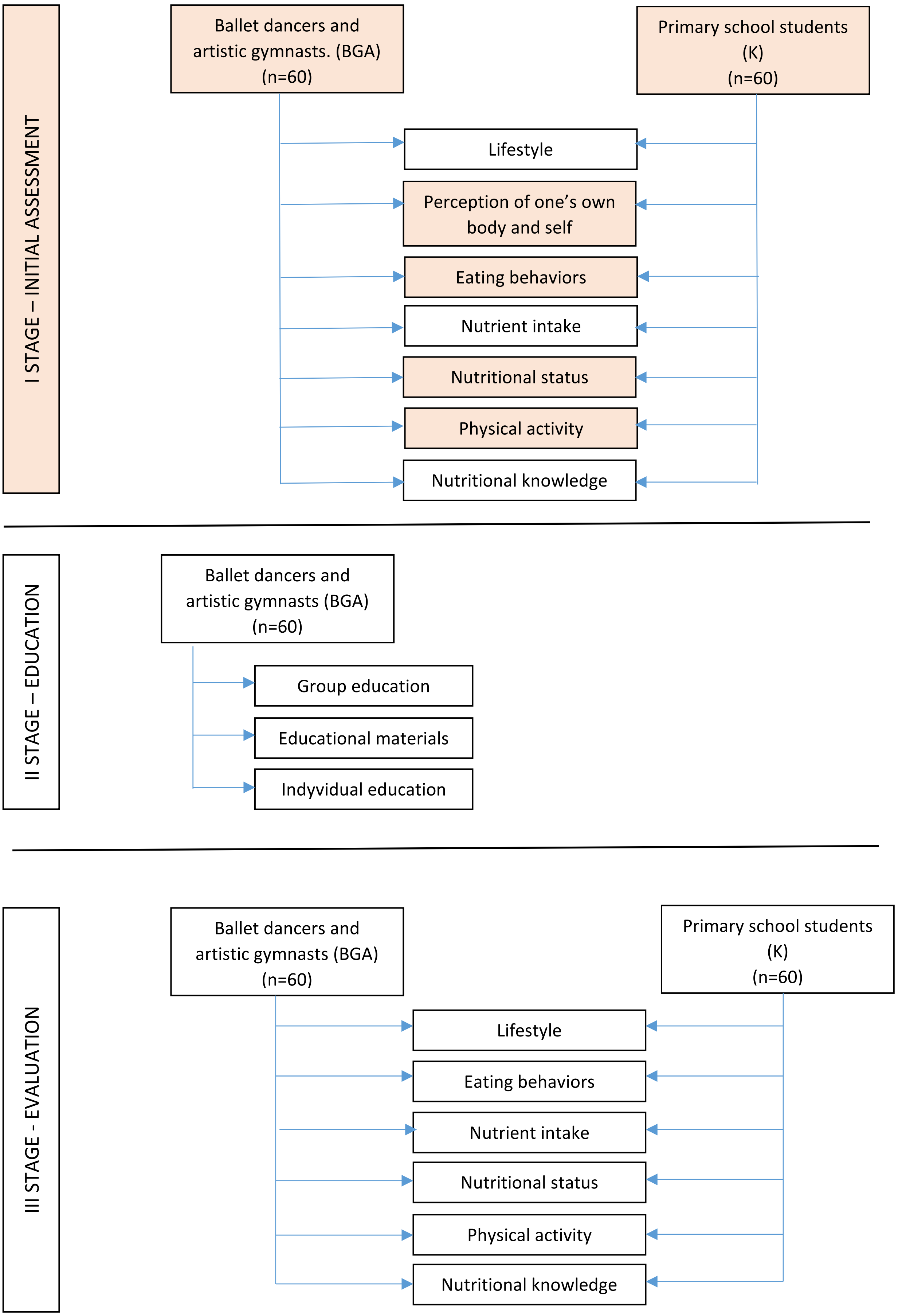

Figure 2. Scheme of the project for assessing the impact of nutritional education on changes in the diet and nutritional status of ballet school students and artistic gymnastics classes.
The stages highlighted in color refer to the content of the results presented in this study.
